# Supplementary material for: G protein coupling and activation of the metabotropic GABAB heterodimer
Source: Nat Commun. 2022 Aug 8;13:4612. doi: 10.1038/s41467-022-32213-3 (PMC9360005; doi:10.1038/s41467-022-32213-3)
Supplement: Supplementary file 1 — Supplementary Information [file 41467_2022_32213_MOESM1_ESM.pdf]

Supplementary Information

G Protein Coupling and Activation of the Metabotropic GABA$_B$ Heterodimer

Moon Young Yang, Soo-Kyung Kim, and William A. Goddard III*

Materials and Process Simulation Center, California Institute of Technology, Pasadena, CA, 91125

***Corresponding author:** wag@caltech.edu or wagoddard3@gmail.com

This document includes:

Supplementary Figure 1. RMSD of the active GABABR heterodimer without GP.

Supplementary Figure 2. The structure of the active GABA$_B$R without PAM.

Supplementary Figure 3. Structural comparison of GABA$_B$R with class A GPCRs.

Supplementary Figure 4. Results of MD simulations for the GABA$_B$-G$_i$P complex structure

Supplementary Figure 5. Sequence alignment of representative class C GPCRs.

Supplementary Figure 6. TM3-6 interactions in the inactive and active states.

Supplementary Figure 7. Result of the metaD simulation for formation of the TM3-Gα5 interaction

Supplementary Figure 8. Results of metaD simulations for Gα opening.

Supplementary reference 1

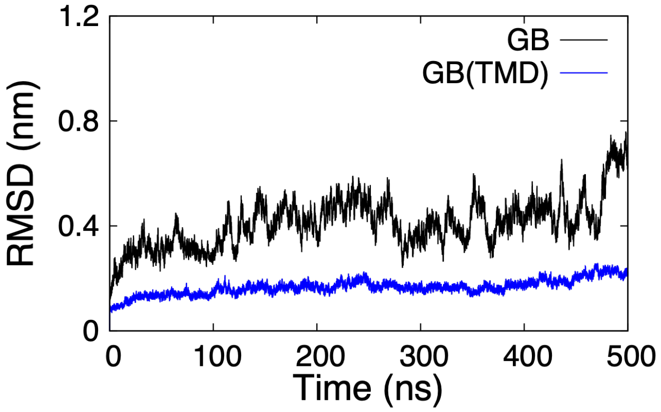

Supplementary Figure 1 | RMSD of the active GABA$_B$R heterodimer without GP. RMSDs were calculated with respect to the initial structure of the simulation.

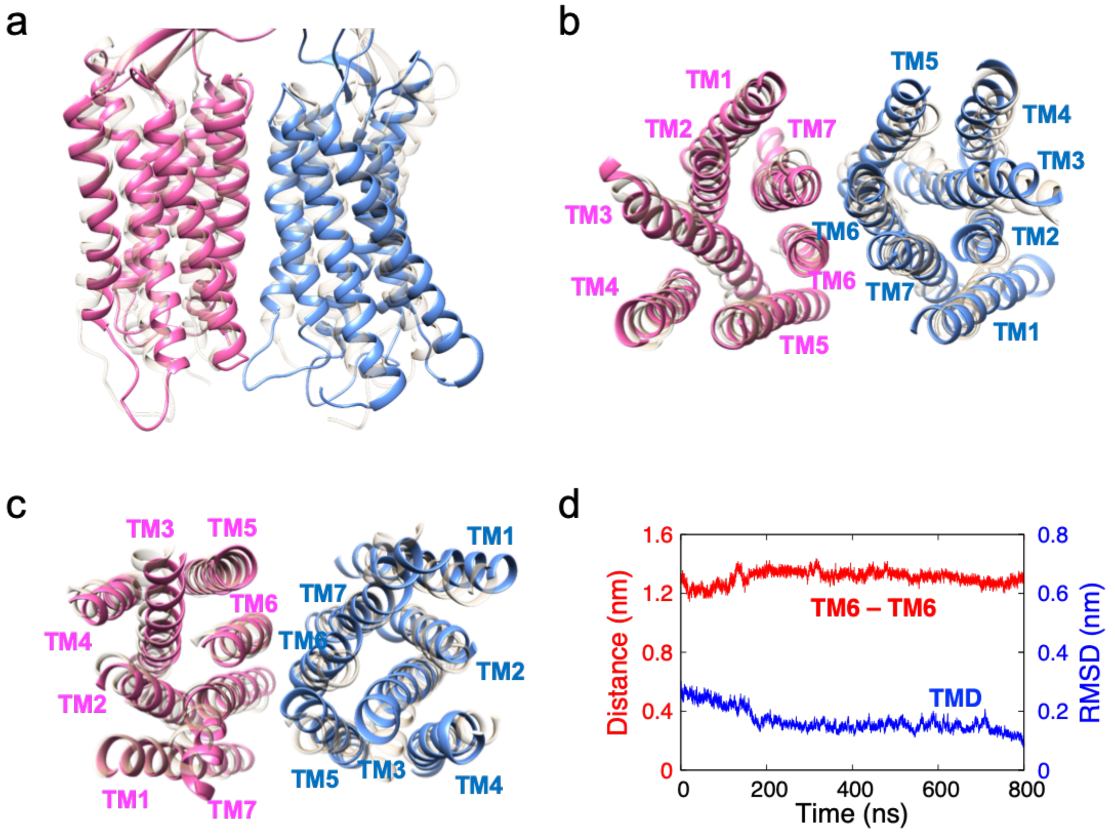

Supplementary Figure 2 | The structure of the active $GABA_BR$ without PAM. (a to c) Comparison of dimeric TMDs interface between the initial (transparent) and the final structures of MD simulation shows that the TM6/TM6 interface is stable during 800 ns MD simulation even without PAM. Here the views are (a) side, (b) top (extracellular side), and (c) bottom (cytoplasmic side), respectively. (d) The distance between GB1 TM6 (the centre of mass of Cα for residues 802-825) and GB2 TM6 (the centre of mass of Cα for residues 689-712) and the RMSD (referenced to the final structure) were measured during the MD simulation.

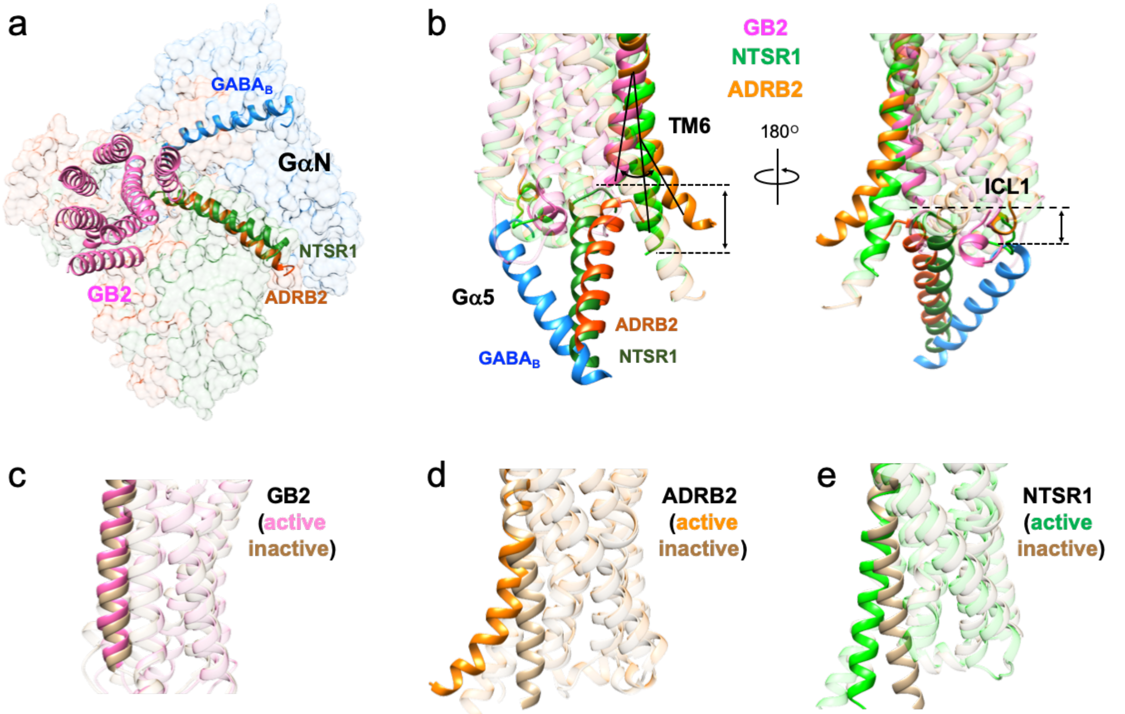

Supplementary Figure 3 | Structural comparison of GABA$_B$R with class A GPCRs. (a) Relative GαN positions of the Gα subunit with respect to the receptor shows that GABA$_B$R has a distinct GP binding mode compared to typical class A GPCRs, β2 adrenergic receptor (ADRB2) and neurotensin receptor 1 (NTSR1), where only GPs are shown for ADRB2 and NTSR1 for clarity. (b) GABA$_B$R has major differences in TM6 and ICL1 compared to class A GPCRs, resulting in the significantly different binding of the Gα5 helix of the Gα subunit. (c to e) Compared to class A GPCRs, TM6 of GABA$_B$R does not show the outward movement upon the activation. PDB codes: 7EB2 and 7C7S for active and inactive GABA$_B$R, 3SN6 and 6PS6 for active and inactive ADRB2, and 6OS9 and 4BUO for active and inactive NTSR1, respectively.

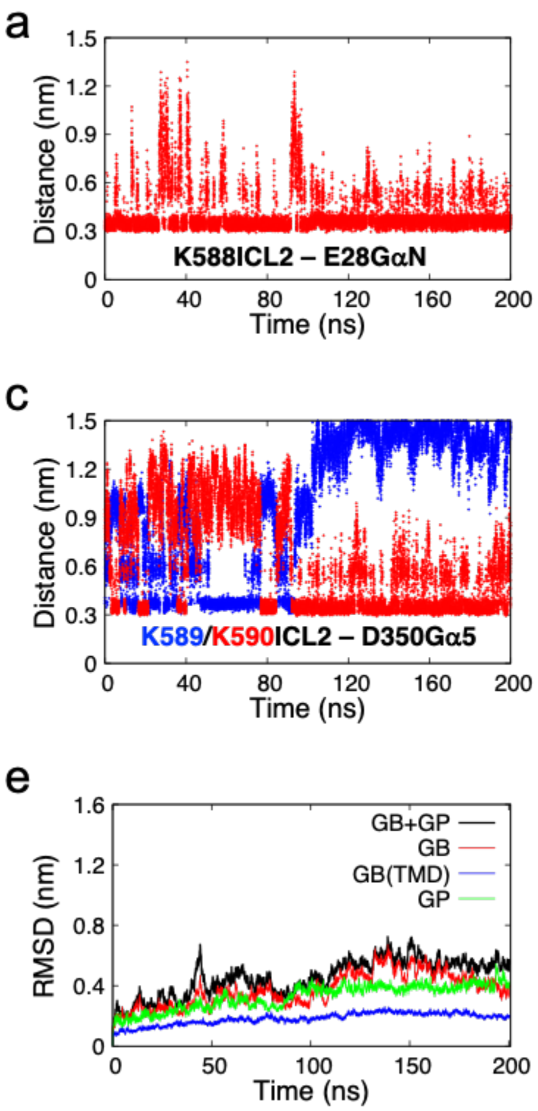

Supplementary Figure 4 | Results of MD simulations for the $GABA_B$-G_iP complex structure. (a to d) $GABA_BR$ forms a stable complex structure with the G_iP *via* multiple salt bridge interactions at the interface. (e) RMSDs (referenced to the initial structure) were measured during the MD simulation.

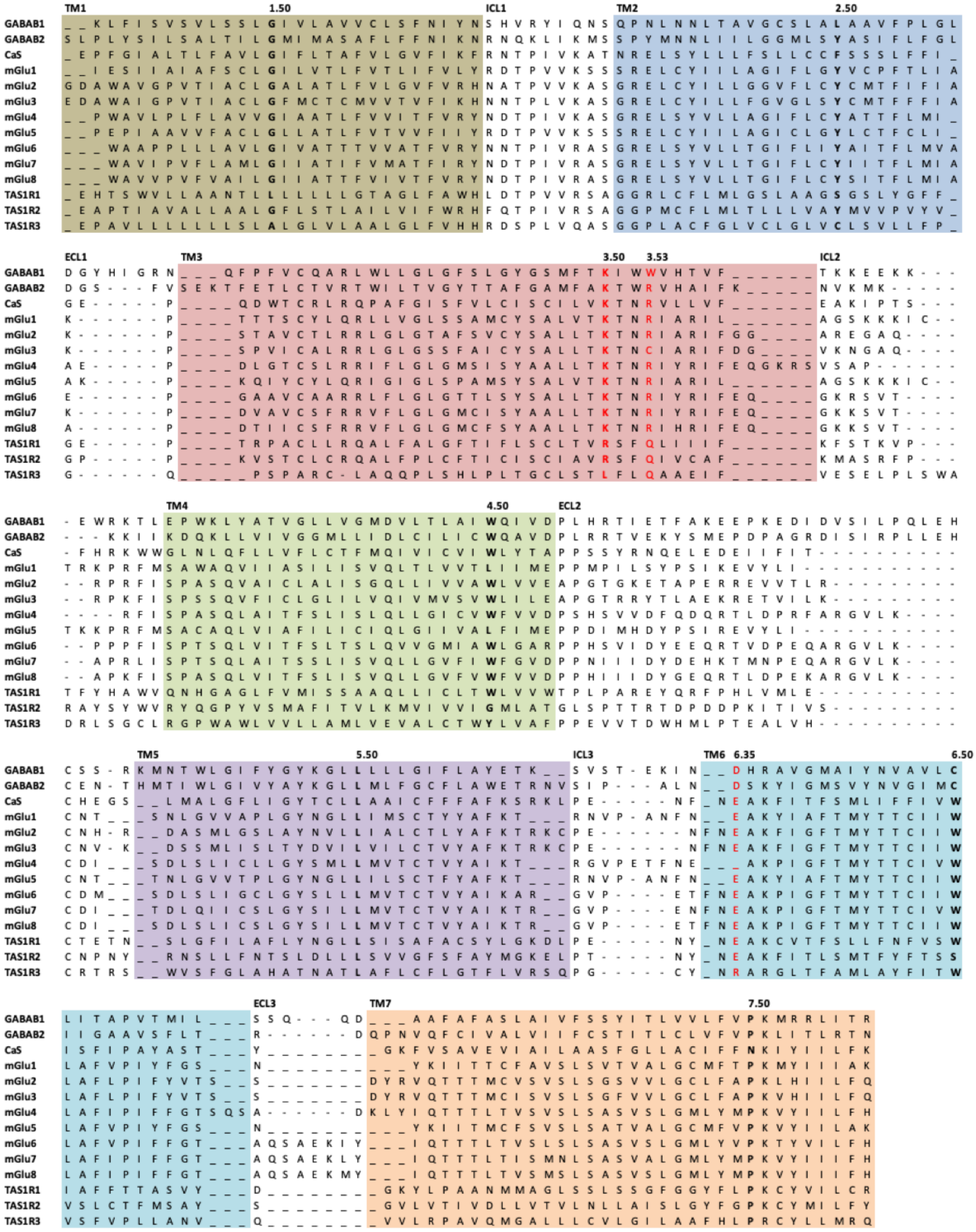

Supplementary Figure 5 | Sequence alignment of representative class C GPCRs.[1]

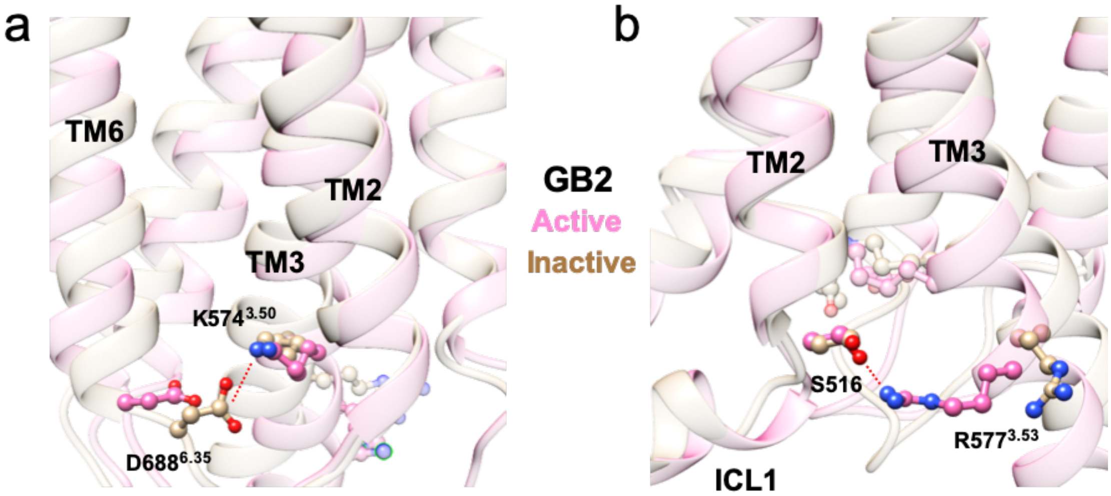

Supplementary Figure 6 | TM3-6 interactions in the inactive and active states. (a) The ionic lock, K574$^{3.50}$-D688$^{6.35}$, is formed only in the inactive conformation, whereas (b) the secondary lock, S516-R577$^{3.53}$, is observed in the active conformation. Here cryo-EM structures were used for the comparisons (PDB codes: 7C7S and 7EB2 for inactive and active conformations of GABA$_B$R, respectively).

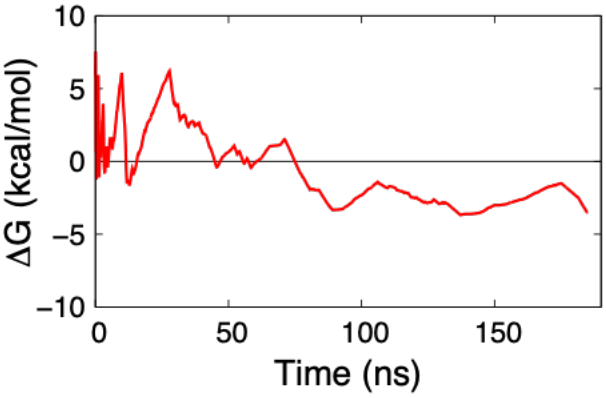

Supplementary Figure 7 | Result of the metaD simulation for formation of the TM3-Gα5 interaction. The change in the free energy difference with time was calculated to monitor the free energy convergence, where a negative value indicates that this reaction is exothermic.

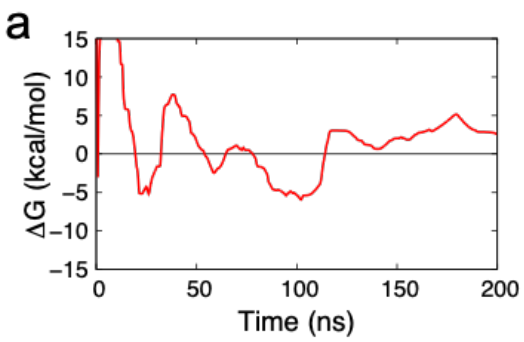

Supplementary Figure 8 | Results of metaD simulations for Gα opening. The change in the free energy differences with time were calculated to monitor the free energy convergence. (a) The negative value indicates that the opening of the Gα subunit is energetically unfavourable when the Gα5 helix is in contact to TM3, (b) however this becomes favourable when the Gα5 helix is deeper inserted and interacts with ICL1.

Supplementary reference

1. Pándy-Szekeres, G. *et al.* GPCRdb in 2018: adding GPCR structure models and ligands. *Nucleic Acids Res.* **46**, D440–D446 (2018).
